# A review and case study of *Rhododendron moulmainense* highlights the feasibility and adaptation of evergreen *Rhododedron* plants to current environmental challenges

**DOI:** 10.3389/fpls.2025.1468526

**Published:** 2025-09-05

**Authors:** Sijia Liu, Peng Zhuang, Zipeng Cai, Yuqing Bai, Jingen Peng, Zaid Khan, Luwen Zhang, Rongsheng Li, Jinchang Yang, Hongyue Cai, Lijuan Xie

**Affiliations:** ^1^ College of Architectural Engineering, Shenzhen Polytechnic University, Shenzhen, China; ^2^ Forest Resources Research Center, Research Institute of Tropical Forestry, Chinese Academy of Forestry, Guangzhou, China; ^3^ Administrative Office of Wutong Mountain National Park, Shenzhen, China

**Keywords:** *Rhododendron moulmainense*, climate change, stress response, mycorrhizal fungi, breeding and cultivation

## Abstract

Alpine rhododendrons have high ecological, ornamental, and recreational value due to its colourful flowers and tall trees, and making it a promising candidate for urban gardens. However, its long growth cycle and lack of adaptation to low altitude environments often result in leaf burning and weak plant growth, hindering its widespread use in urban gardens. Moreover, the existing literature often fails to present key information on propagation techniques and low altitude acclimatisation of alpine rhododendrons in a clear and concise manner. To tackle this issue, we used the example of the alpine evergreen azalea, *Rhododendron moulmainense*, which grows in the southernmost part of the latitude. We conducted a comprehensive review of research advances in the evolutionary status of rhododendrons, mycorrhizal symbiosis, flower bud differentiation, environmental adaptation, and reproduction. By integrating various aspects, this review offers valuable insights into the domestication of alpine rhododendron at low altitudes and proposes solutions to address their environmental adaptation, with the aim of promoting their use in urban gardens and fully utilising their role in ecological stabilisation.

## 1 Introduction of the *Rhododendron* genus

*Rhododendron* L. is the largest genus in the Ericaceae family (Tian, 2011), with over 1000 species of *Rhododendron* plants worldwide. China is recognized as the primary center of origin and diversity for Rhododendron, hosting approximately 570 species, of which around 400 are endemic (Zhuang, 2012; Zhang C, 2022). The alpine rhododendron occupies an important position in the genus *Rhododendron*. The alpine rhododendron refers to a horticultural variety of evergreen rhododendrons, mainly belonging to the subgenus: *Rhododendron*, *Hymenanthes*, and *Azaleastrum*. These evergreen shrubs or small trees are native to high-altitude regions and have undergone centuries of hybridization (Xu et al., 2020). They are mainly distributed across alpine and subalpine zones, where they play a vital role in maintaining ecosystem balance. With their beautiful shape and bright colors, alpine rhododendrons are also valued as ornamental resources in urban landscaping and greening initiatives (Tang et al., 2023). However, due to the strict climatic conditions in high mountains, the diversity index of alpine rhododendron communities is low, making them simple, unstable and weakly resistant to disturbance (Li, 2006; Liu J. et al., 2012; Li et al., 2015).

As shallow-rooted species, the alpine rhododendron has co-evolved with rhizosphere endophytic fungi to form specialized symbiotic systems known as ericoid mycorrhizae (ERM) (Wei et al., 2022). The ERM can enhance the nutrient absorption efficiency of rhododendrons, and improve their stress resistance (Chen et al., 2017; Kerley and Read, 1997; Wei et al., 2022), and are of great significance for the growth and development of as well as the stability of alpine ecosystems.

Rhododendron species exhibit considerable taxonomic complexity, with intricate phylogenetic relationships that are critical to unravel for breeding and genetic improvement. Currently, four major molecular identification techniques are widely employed to investigate the phylogenetic affiliations within the genus: (1) Simple Sequence Repeat (SSR), which offers advantages such as ease of use, minimal DNA requirement, high polymorphism, and co-dominant inheritance (Dikshit et al., 2007); (2) Randomly Amplified Polymorphic DNA (RAPD), which offers advantages such as low DNA consumption, relaxed DNA purity standards, non-radioactive isotopes, simplicity of operation, and heightened sensitivity (Wei et al., 1997; Wen et al., 2004); (3) Restriction-site Associated DNA Sequencing (RAD-seq), providing uniform genome coverage and enabling the identification of thousands of single nucleotide polymorphism (SNP) loci without the need for PCR or electrophoresis (Li et al., 2019); (4) Inter-simple Sequence Repeat (ISSR), valued for its simplicity, independence from known gene sequences, precision, and reproducibility (Dasgupta et al., 2015; Kochieva et al., 2002; Yoshiaki et al., 2002).

These molecular identification methods have been widely applied to various rhododendron species (Liu Y. et al., 2012; Schwemm et al., 2014). Specifically, ISSR, SSR, and RAD-seq have been instrumental in elucidating the phylogenetic position of *Rhododendron moulmainense* (*R. moulmainense*) within the *Rhododendron* genus. Studies have revealed close genetic relationships between *R. moulmainense* and species such as *R. championiae*, *R. stamineum*, *R. hancockii*, *R. latoucheae*, *R. ovatum* (*Lindley*), and *R. vialii*, all members of the subgenus *Azaleastrum* (Li et al., 2019; Liao et al., 2018; Luo et al., 2017). This genetic proximity highlights *R. moulmainense*’s evolutionary placement and its relevance for germplasm introduction, interspecific hybridization, and cultivar development (**Table 1**). Such insights not only inform taxonomic classification but also provide a strategic framework for breeding programs aimed at improving floral traits, heat tolerance, and ornamental longevity. As a genetically distinct species with notable aesthetic appeal and broad ecological adaptability, *R. moulmainense* emerges as a promising candidate for domestication and landscape integration, bridging conservation priorities with horticultural innovation.

**Table 1:** Analysis of the affinity relationships of *Rhododendron moulmainense.*.

Molecular technology	Analytical method	Subgenus	Subgroup		Closer relatives of Rhododendron	References
			*Section Choniastrum*	*Section Azaleastrum*		
ISSR	UGPMA cluster analysis	*Azaleastrum*	*Rhododendron hancockii*, *Rhododendron stamineum*, *Rhododendron championiae*, *Rhododendron latoucheae*, *Rhododendron moulmainense*	*Rhododendron ovatum (Lindley)*, *Rhododendron vialii*	*Rhododendron ovatum (Lindley)*, *Rhododendron vialii*	(Li et al., 2019)
RAD-seq	Maximum likelihood parentage analysis of GTP+CAT model				*Rhododendron hancockii*, *Rhododendron stamineum*	(Liao et al., 2018)
SSR	UGPMA cluster analysis				*Rhododendron championiae*, *Rhododendron ovatum (Lindley)*, *Rhododendron latoucheae*	(Luo et al., 2017)

Due to the strict growth environment requirements of alpine rhododendrons, the varieties successfully introduced into urban landscaping remain limited. *R. moulmainense*, as a representative species of alpine rhododendron within the subgenus *Azaleastruma*, holds particular significance for evolutionary and adaptive studies in the genus *Rhododendron* (Wang et al., 2021). Therefore, research on *R. moulmainense* carries representative value for the introduction and promotion of high-altitude rhododendron species. In this review, we comprehensively summarize recent advances in the study of *R. moulmainense*, including its distribution and ecological characteristics, photosynthetic physiology, root system and microbial diversity, flower bud differentiation, stress resistance, and cultivation and reproduction techniques. By synthesizing these findings, we aim to provide a scientific basis for the development and utilization of *R. moulmainense*. As a model species within the genus, *R. moulmainense* offers valuable insights into the introduction and domestication of alpine rhododendrons, with broad application potential in ecological restoration and urban landscaping.

## 2 Distribution and ecological requirements of *Rhododendron moulmainense*

### 2.1 Geographic distribution and morphological characteristics of *Rhododendron moulmainense*

**Figure 1:**
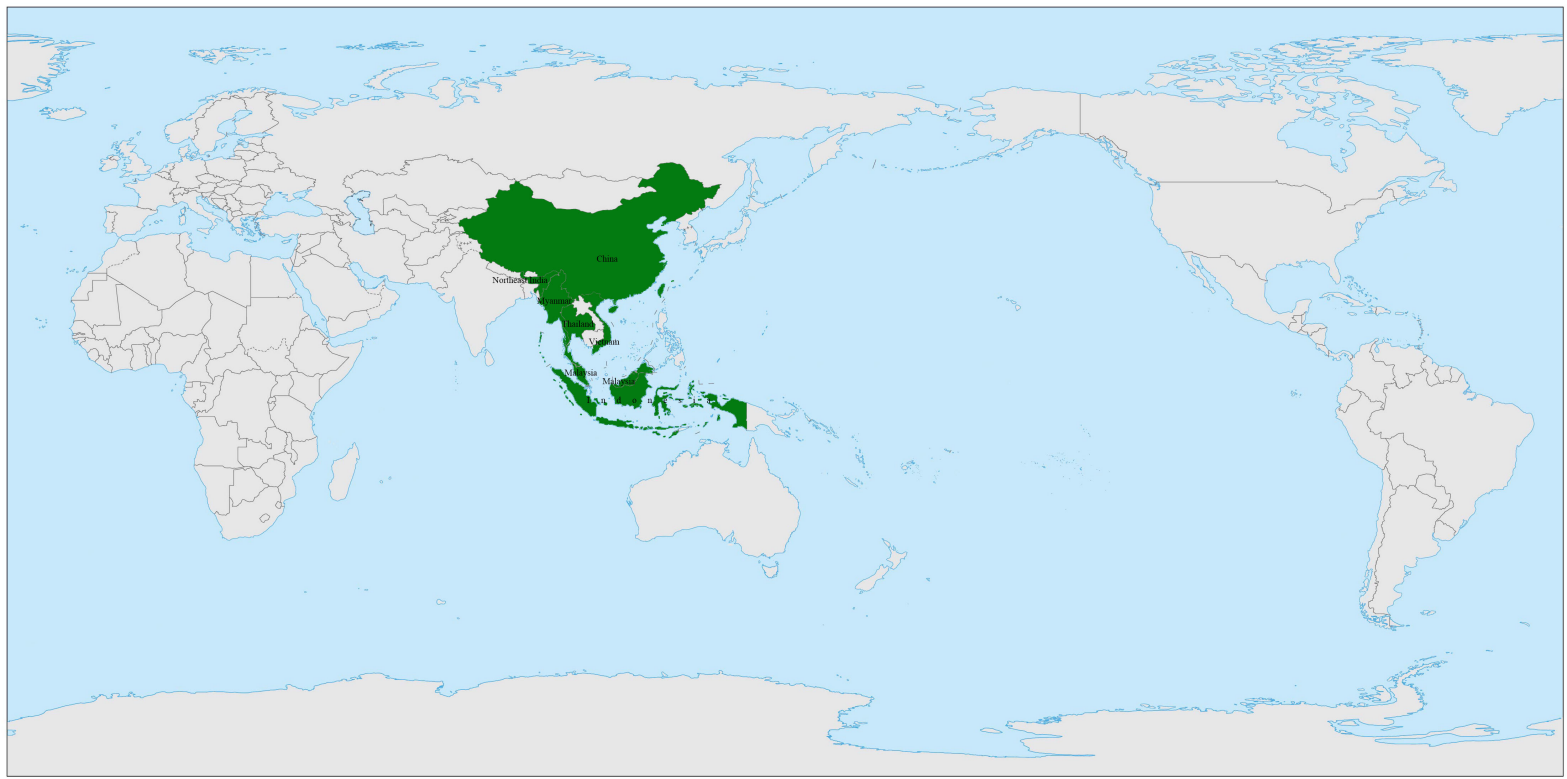
The distribution of *Rhododendron moulmainense* worldwide. The *Rhododendron moulmainense* is distributed in Asia, specifically in China, Vietnam, Myanmar, Thailand, Malaysia, Indonesia, and the northeastern region of India. This map is based on the standard map with the approval number GS (2016)1665 downloaded from the Ministry of Natural Resources Standard Map Service website (https://bzdt.ch.mnr.gov.cn/), and the base map has not been modified.

**Figure 2:**
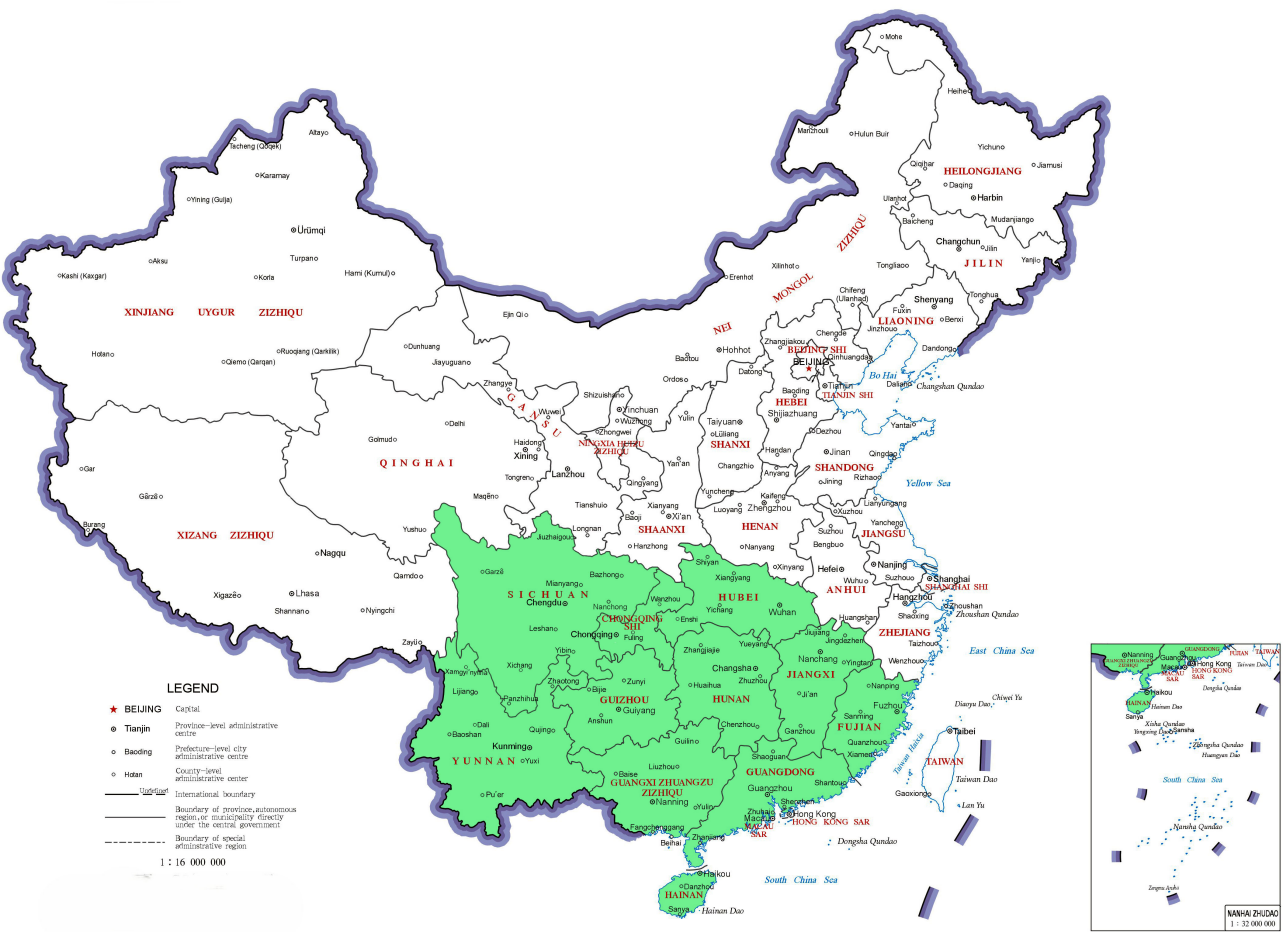
The distribution of *Rhododendron moulmainense* in China. *Rhododendron moulmainense* is found in various regions within China, including Sichuan Province, Hubei Province, Hunan Province, Jiangxi Province, Fujian Province, Guangdong Province, Guangxi Zhuang Autonomous Region, Hainan Province, Guizhou Province, Yunnan Province, Chongqing Municipality, and Hong Kong Special Administrative Region. This map is based on the standard map with the approval number GS (2019) 1686 downloaded from the Ministry of Natural Resources Standard Map Service website (https://bzdt.ch.mnr.gov.cn/), and the base map has not been modified.

*R. moulmainense* is predominantly found in high-altitude environments ranging from 700 to 1400 meters, with its natural range spanning several Asian countries, including China, Vietnam, Malaysia, Indonesia, Myanmar, Thailand, and northeastern India (Flora of China Editorial Committee, 2005; Song et al., 2015) (**Figure 1**). Within China, *R. moulmainense* exhibits its widest distribution, occurring across numerous provinces, municipalities, and special administrative regions such as Guangdong, Guangxi, Hainan, Jiangxi, Fujian, Hunan, Hubei, Sichuan, Guizhou, Yunnan, Chongqing, and Hong Kong (Huang et al., 2010; Ng and Corlett, 2003; Wang et al., 2017; Xu, 2013; Editorial Board of Flora of China, Chinese Academy of Sciences, 1994) (**Figure 2**).

Morphologically, *R. moulmainense* typically grows to a height of 3-7 meters, with some individuals reaching up to 10 meters. It is characterized by a dense canopy, profuse branching, and a semi-open crown structure (Xu, 2013). The bark is brown, and young shoots initially bear hairs that shed over time, leaving the plant glabrous. Its opposite, leathery leaves are broad and smooth, transitioning from yellowish-green to dark green as they mature. The leaves are elliptical-lanceolate or broadly oblong-lanceolate in shape, with pointed or acute apices and cuneate bases, and are usually clustered at branch tips (Kang et al., 2009; Li and Jia, 2012). The flowers of *R. moulmainense* are borne in terminal clusters of 3 to 8, with corollas that are funnel-shaped to bell-like. Floral coloration ranges from white and off-white to pink and light pink. The flowers are distinguished by a hairless calyx and pedicel, and a petal marked with a brownish-yellow spot at the throat. Flowering occurs from January to March, peaking in March when the blooms begin to fade. The fruit is a slender, cylindrical capsule with six prominent ribs (Kang, 2009; Xu, 2013).

Ecologically, *R. moulmainense* prefers well-aerated, well-drained acidic soils (pH 4.5-6.0) rich in humus. It thrives in sunlight but is sensitive to excessive light intensity (Kang et al., 2009; Zhang et al., 2023). The species flourishes in cool, humid conditions and exhibits low tolerance to heat stress (Li and Jia, 2012; Liu et al., 2024).

With its striking floral display and ecological adaptability, *R. moulmainense* holds significant ornamental and ecological value (Huang et al., 2010). This is particularly evident on Wutong Mountain in Shenzhen, where its vibrant blossoms contribute to the mountain’s seasonal grandeur. The interplay between the plant and its natural environment creates a scenic landscape reminiscent of traditional ink paintings, making *R. moulmainense* a cherished symbol and popular attraction for local residents (Liu et al., 2015a).

### 2.2 Photosynthetic growth characteristics of *Rhododendron moulmainense*

Light is a critical environmental factor influencing the photosynthetic efficiency, growth and development of plants (Zhang et al., 2005). The intensity of light directly influences plant photosynthesis by serving as the primary energy source for the assimilation of carbon, thereby driving the formation of dry matter and biomass accumulation (Pires et al., 2011; Wen et al., 2018). However, excessive light may lead to photo-inhibition, reducing the photosynthetic rate and ultimately impeding overall plant growth (Shi et al., 2022). Therefore, photosynthesis, as a fundamental metabolic process, exerts a central role in regulating plant physiological performance and developmental trajectories.

In a comparative study of four Rhododendron species (*R. moulmainense*, *R. latoucheae*, *R. fortunei*, and *R. rivulare*), *R. moulmainense* demonstrated a distinctive hierarchy of physiological and ecological factors affecting its net photosynthetic rate, ranked as follows: stomatal conductance > air concentration > air humidity > transpiration rate > air temperature > photosynthetically active radiation (Liao, 2011). Further investigations revealed that while low light intensity suppresses seedling growth, it paradoxically enhances photosynthetic efficiency, whereas excessive light impairs both growth and photosynthetic performance in *R. moulmainense* (Wei and Zhuang, 2014). When compared with other species such as *R. eudoxum* and *R. simsii*, *R. moulmainense* exhibited markedly higher light energy utilization efficiency, reflecting its adaptive photosynthetic traits under specific light regimes (Zhang et al., 2012). The latest research showed that *R. moulmainense* grows better under semi-shaded condition than under full-light condition, which promoting the number and thickness of new shoots and the formation of flower buds. Moreover, the chlorophyll content under semi-shaded condition is higher than that under full-light condition (Zhang et al., 2023).

Given the dominant influence of stomatal conductance on the net photosynthetic rate of *R. moulmainense* (Liao, 2011), strategic enhancement of stomatal regulation presents a promising avenue for optimizing carbon assimilation. Moreover, meticulous control of cultivation light conditions is imperative, as both insufficiency and excess can destabilize photosynthetic equilibrium. Improving light energy conversion efficiency may therefore be a key strategy for promoting growth and maintaining physiological resilience in *R. moulmainense*.

Despite these findings, significant knowledge gaps remain regarding the mechanistic basis for the photosynthetic efficiency observed in *R. moulmainense*. For instance, the roles of chloroplast ultrastructure, plastoglobule dynamics, and retrograde signaling in mediating light adaptation responses have not yet been systematically elucidated (Arzac et al., 2022; Zhu, 2016). Moreover, integrating multi-omics approaches such as transcriptomics and metabolomics would enable deeper insight into the regulatory networks underlying light acclimation. Such investigations may also reveal connections to stress response pathways, offering new perspectives on how light-related physiological traits contribute to drought tolerance or temperature adaptation.

### 2.3 Diversity and functional roles of mycorrhizal fungi in *Rhododendron moulmainense*

Plant growth and development are influenced by both intrinsic physiological processes and extrinsic ecological conditions. Among these, microbial diversity, particularly soil microorganisms, plays an essential role in enhancing plant health, metabolism, and stress resilience (Yang et al., 2017). Soil microbial communities are foundational components of soil ecosystems, directly impacting soil fertility, nutrient cycling, and plant productivity (Duineveld et al., 2001; Wang et al., 2019). The plant rhizosphere is typically inhabited by diverse microbial populations, and plant roots can develop a mutually beneficial symbiotic relationship with soil fungi, known as mycorrhiza. These associations contribute to niche differentiation and expand the ecological adaptability of host plants (Gerz et al., 2018; Ulrich et al., 2008).

Isolation and identification of mycorrhizal fungi commonly involve culturing and purifying fungal mycelia, followed by morphological characterization and molecular analysis. Standard media used for fungal cultivation include MMN, MEA, MA, PSA, Czapek Dox Agar, and PDA (Wu, 2022; Yuan, 2022). Mycelium purification is usually accomplished using PDA, followed by the isolation of a single purified strain. The morphology and color of the individual mycelium were documented for comparison with known fungi. Moreover, the purified mycelium is typically subjected to DNA extraction and gene amplification, with species identification conducted by sequence comparison against the NCBI database (Mu et al., 2021). Subsequently, the mycelium was introduced into sterile seedlings by utilizing the root system of sterile seedlings, and followed by tissue staining (e.g., with Trypan blue) and microscopic examination for root colonization (Cai et al., 2021).

**Figure 3:**
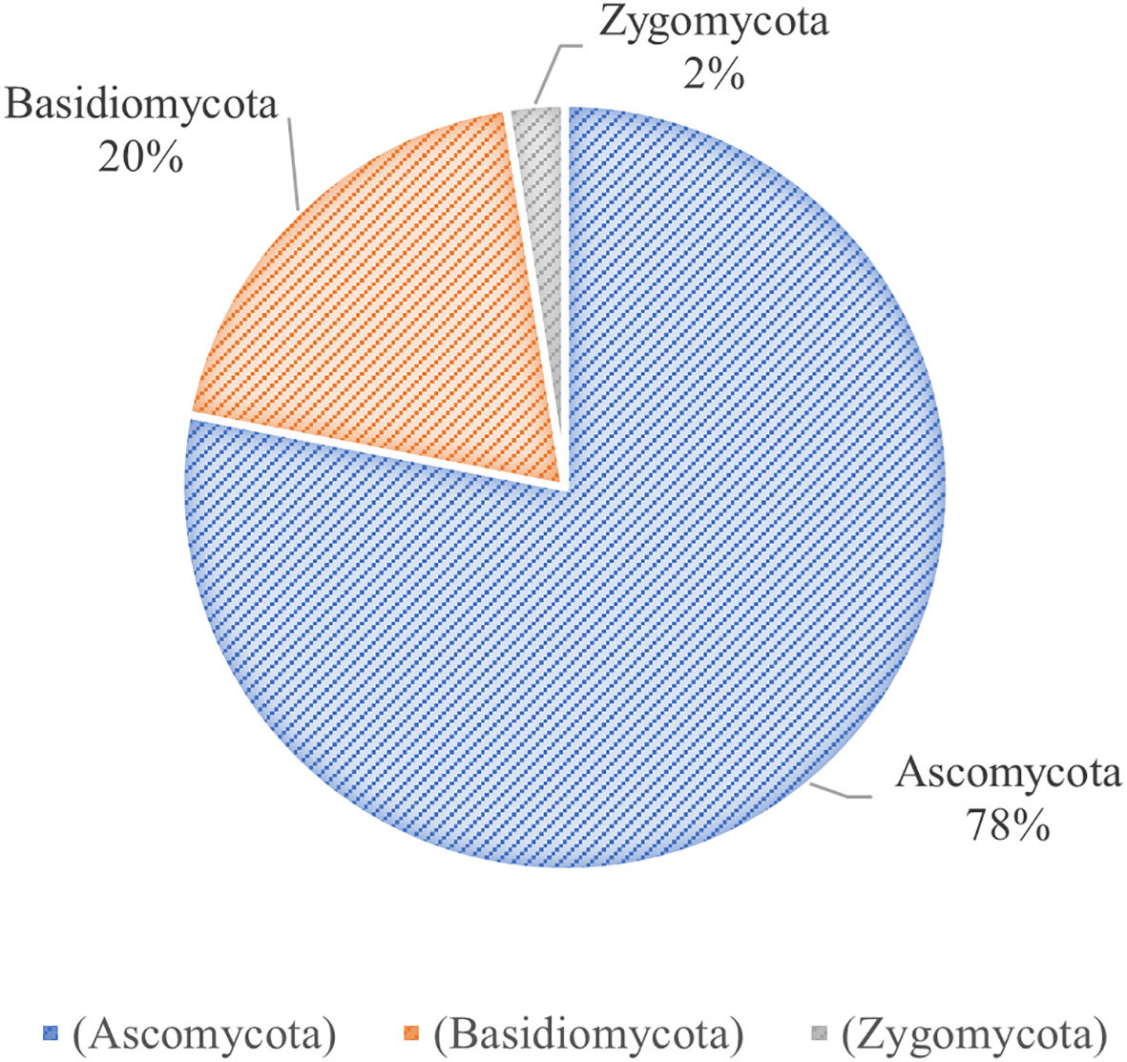
Classification of mycorrhizal fungi in *Rhododendron moulmainense*. The mycorrhizal fungi species in the root system of *Rhododendron moulmainense* belong to the subkingdoms Ascomycota, Basidiomycota, and Zygomycota.

Rhododendrons serve as the main hosts of ericoid mycorrhizas (ERM), which is commonly found in different ecosystems. The ability of rhododendrons to thrive on diverse substrates, such as acidic sandy soils and moist coarse humus, is attributed to the symbiotic relationship with ERM (Perotto et al., 2002). Therefore, unraveling the association between mycorrhizal fungi and *R. moulmainense* development is crucial for improving seedling survival and establishment. Current research on the relationship between *R. moulmainense* and microorganisms mainly focuses on root microbiome diversity and its regulation of *R. moulmainense* growth. The root fungi of *R. moulmainense* are diverse, and many specific bacterial and fungal colonies are distributed around *R. moulmainense* roots (Peng et al., 2022). Moreover, different tree vigor levels have significant differences in the microbial community structure of *R. moulmainense* rhizosphere soil and root, and fungal community structure is closely related to tree vigor differences (Gong et al., 2021; Zhuang et al., 2025). Through direct gene sequencing and mycelial culture, 40 fungal species (**Table 2**) spanning Ascomycota, Basidiomycota, and Zygomycota have been identified in its rhizosphere (**Figure 3**) (Fehrer et al., 2019; Liao et al., 2016; Liu et al., 2015b; Perotto et al., 2018; Vohnik, 2020).

**Table 2:** The mycorrhizal fungi species identified from *Rhododendron moulmainense*.

Number	Named Fungal species	GenBank Serial number	Blast Similar fungi	Similar Fungal serial numbers	References
1	*Agaricales* sp*1*	KU550104	*Hemimycena* sp.	HQ604775	(Liao et al., 2016)
2	*Agaricales* sp*2*	KU550105	*Mycena* sp.	KP012834	(Liao et al., 2016)
3	*Agaricomycetes* sp.	KU550106	*Cyptotrama asprata*	DQ097355	(Liao et al., 2016)
4	*Chaetosphaeriaceae* sp.	KU550107	*Chaetosphaeria* sp.	AY781219	(Liao et al., 2016)
5	*Cladophialophora* sp.	KU550108	*Cladophialophora* sp.	AB986333	(Liao et al., 2016)
6	*Helotiaceae* sp*1*	KU550109	*Rhizoscyphus ericae*	AB847029	(Liao et al., 2016)
7	*Helotiaceae* sp*2*	KU550130	*Hymenoscyphus ericae*	AY394684	(Liao et al., 2016)
8	*Helotiales* sp*1*	KU550131	*Helotiales* sp.	EU639688	(Liao et al., 2016)
9	*Helotiales* sp*2*	KU550110	*Helotiales*	KC019885	(Liao et al., 2016)
10	*Helotiales* sp*3*	KU550111	*Helotiales* sp.	JX852326	(Liao et al., 2016)
11	*Helotiales* sp*4*	KU550112	*Scytalidium* sp.	HQ631037	(Liao et al., 2016)
12	*Herpotrichiellaceae* sp*1*	KU550113	*Herpotrichiellaceae* sp.	AB847033	(Liao et al., 2016)
13	*Herpotrichiellaceae* sp*2*	KU550114	*Herpotrichiellaceae* sp.	KF359595	(Liao et al., 2016)
14	*Hyaloscyphaceae* sp*1*	KU550115	*Lachnum virgineum*	JQ272454	(Liao et al., 2016)
15	*Hyaloscyphaceae* sp*2*	KU550116	*Lachnum* sp.	KJ529001	(Liao et al., 2016)
16	*Hypocreales* sp.	KU550117	*Neonectria lugdunensis*	FJ000394	(Liao et al., 2016)
17	*Lachnum* sp*1*	KU550118	*Lachnum* sp.	KJ817288	(Liao et al., 2016)
18	*Lachnum* sp*2*	KU550119	*Lachnum* sp.	FJ440910	(Liao et al., 2016)
19	*Mortierella* sp.	KU550120	*Mortierella biramosa*	JX976094	(Liao et al., 2016)
20	*Oidiodendron* sp.	KU550121	*Oidiodendron citrinu*	NR_111033	(Liao et al., 2016)
21	*Pezizomycotina* sp.	KU550122	*Pezizomycotina*	JF519200	(Liao et al., 2016)
22	*Phialocephala fortinii*	KU550123	*Phialocephala fortini*	NR_103577	(Liao et al., 2016)
23	*Rhytismataceae* sp.	KU550124	*Rhytismataceae* sp.	JQ272405	(Liao et al., 2016)
24	*Russula* sp.	KU550125	*Russula favrei*	KC581298	(Liao et al., 2016)
25	*Russulaceae* sp.	KU550126	*Russula vesca*	KM085395	(Liao et al., 2016)
26	*Sebacinaceae* sp.	KU550127	*Sebacina* sp.	AB831790	(Liao et al., 2016)
27	*Thelephoraceae* sp.	KU550128	*Tomentella*	GU553375	(Liao et al., 2016)
28	*Trechisporales* sp.	KU550129	*Trechispora farinace*	EU909231	(Liao et al., 2016)
29	*——*	——	*Mollisia cinerea*	JF514855	(Liu et al., 2015b)
30	*——*	——	*Phialocephala* sp.	KF156325	(Liu et al., 2015b)
31	*——*	——	*Uncultured Helotiales* sp.	KF498574.1	(Liu et al., 2015b)
32	*——*	——	*Aspergillus sydowii*	AY373869	(Liu et al., 2015b)
33	*——*	——	*Gloeotinia temulenta*	DQ235697	(Liu et al., 2015b)
34	*——*	——	*Bionectria ochroleuca*	EU273558	(Liu et al., 2015b)
35	*——*	——	*Fusarium oxysporum*	JF439472	(Liu et al., 2015b)
36	*——*	——	*Sordariomycetes* sp.	AB847034	(Liu et al., 2015b)
37	*——*	——	*Ascomycota* sp.	HQ608112	(Liu et al., 2015b)
38	*——*	——	*Paecilomyces javanicus*	AB099944	(Liu et al., 2015b)
39	*——*	——	*Soil fungal* sp.	EU076958	(Liu et al., 2015b)
40	*——*	——	*Neofusicoccum australe*	KF702388	(Liu et al., 2015b)

Numbers 1-28 representing the species that have already been named and identified.

In terms of the biological regulation of *R. moulmainense* growth, researches have indicated that the root system of this plant species can be stimulated by Ascomycota. The research discovered that after inoculating the root system of *R. moulmainense* with seven different fungi, the effects on the growth of *R. moulmainense* varied among the different fungi, but all enhanced the activity of resistance enzymes Phenylalanine ammonia lyase (PAL) and Lipoxygenase (LOX) in *R. moulmainense*. Among them, the fungi *Bionectria ochroleuca*, *Aspergillus sydowii*, and *Paecilomyces javanicus* showed better promoting effects on the growth of *R. moulmainense* (Zhou et al., 2017a). Further experiments demonstrated that inoculation with dark-septate endophytes (*Phialocephala fortinii*) and *Aspergillus sydowii* significantly improved the growth of 1-year-old and 1.5-year-old *R. moulmainense* seedlings, with the most notable enhancement observed in 1-year-old seedlings treated with *Phialocephala fortinii* (Zhou et al., 2017b). In addition to promoting growth, *Phialocephala fortinii* was found to confer superior drought resistance compared to *Aspergillus sydowii* (Hong et al., 2016). Moreover, inoculating with *Phialocephala fortiniican* could improve the absorption of nitrogen and phosphorus in *R. moulmainens*e, and also improve its resistance to phosphorus stress (Liu et al., 2015c). In a subsequent research, three strains of *Aspergillus sydowii* that were extracted from the roots of *R. fortunei* and *R. simsii*, as well as *Aspergillus versicolor* obtained from the roots of *R. kwangtungense*, were introduced to *R. moulmainense* seedlings. Additionally, a mixture containing the three strains was used to inoculate the root system of *R. moulmainense* seedlings. While each fungal strain and the mixed inoculum successfully established mycorrhizal symbiosis with *R. moulmainense* seedlings, their effects varied across different substrates in terms of root colonization, seedling height, and biomass accumulation (Song et al., 2015).

**Figure 4:**
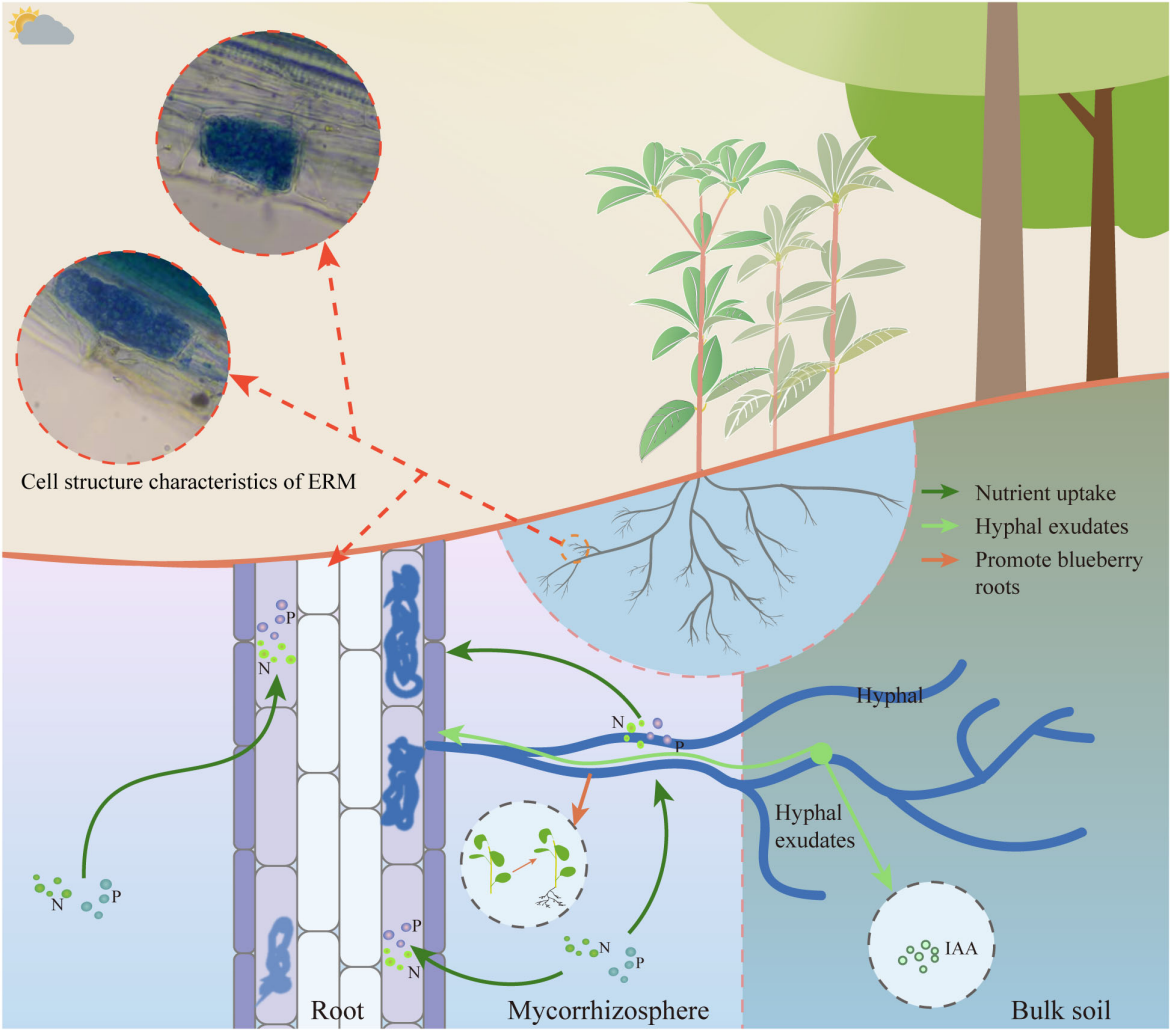
Growth regulation of *R. moulmainense* by ERM fungi. The root system of *R. moulmainense* can absorb nitrogen and phosphorus nutrients during the growth process, and the ERM fungi symbiotic with the root system of *R. moulmainense* can also absorb nitrogen and phosphorus elements transported to the root system of *R. moulmainense*, and it has been shown that the ERM fungi can promote the rooting of the rootless seedlings of blueberries and secrete growth hormones (IAA) to promote the growth of blueberries [94], so perhaps ERM fungi have the same effect on *R. moulmainense*.

These findings underscore the pivotal role of mycorrhizal fungi in supporting *R. moulmainense* growth (Ding et al., 2015; Mu et al., 2021; Cai et al., 2021) (**Figure 4**). Despite promising advances, mechanistic understanding of host-microbe interactions in *R. moulmainense* remains limited. The signaling pathways underlying mycorrhizal recognition, colonization specificity, and host immune modulation are largely unknown (Wei et al., 2020). Moreover, how microbial symbionts influence developmental transitions, such as floral commitment or resource allocation, has not been fully explored. Integrating multi-omics platforms (transcriptomics, metabolomics, metagenomics) with functional assays (e.g., CRISPR-Cas9-mediated genes validation) could elucidate regulatory nodes that govern stress-adaptive traits (Leal et al., 2024; Midgley et al., 2018; Shi et al., 2021; Wang et al., 2021). These insights would not only advance our understanding of rhododendron biology but also inform precision agriculture and ecological restoration strategies in nutrient-poor or climate-sensitive habitats.

### 2.4 Material changes in bud differentiation of *Rhododendron moulmainense*

The differentiation between vegetative (leaf) and reproductive (flower) buds represents a crucial developmental transition in flowering plants, underpinned by dynamic alterations in hormonal and metabolic composition (Guo et al., 2020). In *R. moulmainense*, ring-stripping has been demonstrated to modify nutrient distribution and phytohormone levels, thereby creating favorable physiological conditions for floral bud formation (Kang, 2009). Hormonal profiling in *R. moulmainense* during the differentiation of floral and leaf buds reveals that the top leaves of flower buds exhibit elevated levels of abscisic acid (ABA) and zeatin riboside (ZR), while gibberellic acid (GA_3_) and indole-3-acetic acid (IAA) display relatively higher concentrations in leaf buds. Notably, the ratios of ABA/GA_3_, ABA/IAA, ZR/GA_3_, and ZR/IAA in flower buds are significantly increased, underscoring the critical role of hormonal balance in driving morphological differentiation toward reproductive organogenesis (Xie et al., 2010).

In addition to phytohormonal regulation, metabolic changes, including those involving energy substrates, play an equally pivotal role in bud differentiation (Yap et al., 2008; Shang et al., 2022). During *R. moulmainense* floral initiation, marked shifts were observed in the levels of starch, soluble sugars, and the enzymatic activities of α-amylase and β-amylase. A higher starch-to-soluble sugar ratio was characteristic of flower buds, suggesting starch accumulation acts as a reservoir to support the energy demands of flower formation (Xie et al., 2009a). Measurements of total sugar, nitrogen, soluble protein, and phosphorus in leaf buds during this transition demonstrate the essential role of nutrient availability and metabolic reprogramming in determining bud fate (Xie et al., 2009b).

Although these studies elucidate correlations between hormonal and metabolic profiles and bud fate specification, the underlying regulatory mechanisms remain insufficiently characterized. Particularly, the integration between hormonal signaling and sugar-sensing pathways, such as TOR, SnRK1, or T6P-mediated networks, has not yet been addressed in *R. moulmainense* (Baena-Gonzalez and Hanson, 2017; Kataoka et al., 2004; Wahl et al., 2013). Further exploration into how energy status intersects with hormonal gradients at the molecular level could reveal new insights into the metabolic programming of reproductive (flower) buds. Moreover, linking these findings to broader themes, such as floral phenology under climate variability or resource allocation in stress-adapted perennials, would enhance their relevance within the fields of developmental physiology and ecological adaptation. These avenues could ultimately inform optimized cultivation protocols that enhance flowering efficiency in ornamental or ecological applications.

## 3 Stress responses of *Rhododendron moulmainense*

### 3.1 Environmental adaptation and physiological responses of *Rhododendron moulmainense*

Abiotic stress resistance is a critical adaptive trait that enables plants to survive and grow under unfavorable environmental conditions. In the context of low-altitude urban landscaping, rhododendrons frequently encounter abiotic challenges such as high temperature and drought, two factors that markedly constrain their ecological distribution and ornamental utility (Geng et al., 2019). When subjected to drought stress, plant cells exhibit increased membrane permeability, resulting in ion leakage, elevated electrical conductivity, and compromised selective permeability, ultimately leading to membrane dysfunction (Munns, 2002; Yuan et al., 2024). Although most rhododendrons are naturally adapted to moisture-rich high-altitude habitats, *R. moulmainense* is particularly susceptible to drought conditions due to its limited physiological tolerance (Mayr et al., 2010; Cordero and Nilsen, 2002). However, the molecular and physiological mechanisms by which *R. moulmainense* responds to drought remain poorly elucidated, representing a key gap in current research.

High temperature stress likewise poses significant challenges. Morphological symptoms such as leaf wilting, chlorosis, and stunted growth are immediate indicators of thermal damage (Zheng, 2004). At the cellular level, heat stress disrupts metabolic homeostasis, induces lipid peroxidation, and stimulates reactive oxygen species (ROS) accumulation, processes that can be mitigated by the activation of antioxidant defense systems (Sun, 2015). Studies have indicated that in rhododendron, heat stress triggers substantial increases in ROS levels, osmolyte concentrations, and activities of antioxidant enzymes, all of which contribute to alterations in physiological metabolism (Pan et al., 2022). Additionally, temperature fluctuations interfere with bud differentiation and floral development, and *R. moulmainense* can tolerate temperatures up to 35°C, it struggles to survive at 42°C or higher (Ding, 2016; Liu et al., 2024; Xian and Chen, 2015). Comparative analysis across five rhododendron species revealed that *R. moulmainense* possesses superior heat resistance relative to *R. mucronatum*, *R. molle*, *R. chihsinianum*, and *R. rubiginosum*, suggesting inherent physiological advantages in coping with thermal stress (Wang et al., 2011). Exogenous application of stress-mitigating agents has shown promise in enhancing abiotic stress tolerance. For instance, foliar spraying with 90 mg/L chitosan improves heat resistance in *R. moulmainense* by increasing soluble sugar accumulation and modifying cellular ultrastructure (Ding et al., 2015).

Recent transcriptomic and small RNAome studies have further revealed molecular-level adaptations in *R. moulmainense* under heat stress. Protein folding pathways are rapidly activated to maintain proteostasis, while alternative splicing mechanisms modulate long-term gene expression patterns. Weighted Gene Co-expression Network Analysis (WGCNA) identified key regulatory modules involved in maintaining ROS equilibrium and stress signal transduction (Liu et al., 2024). This study constitutes the first molecular perspective on heat-responsive gene regulation in *R. moulmainense*, providing valuable candidate loci for future genetic engineering aimed at enhancing thermal resilience in alpine evergreen azaleas.

These findings underscore that stress resistance in *R. moulmainense* is not only dependent on physiological adaptations but also increasingly understood through molecular frameworks that integrate hormone signaling, redox balance, and transcriptomic plasticity. However, key questions remain. The crosstalk between abiotic stress-responsive pathways and developmental regulation, such as how drought or heat affects flowering time, stomatal dynamics, and root exudate composition, is still underexplored. Moreover, the role of epigenetic modifications and non-coding RNAs in sustaining stress memory and resilience across developmental stages warrants further investigation (Chang et al., 2020; Imaduwage and Hewadikaram, 2024). Addressing these gaps will be crucial for optimizing the growth and landscape performance of *R. moulmainense* in increasingly volatile climatic scenarios.

### 3.2 Disease and pest threats in *Rhododendron moulmainense*

Biotic stresses, particularly diseases and pests, pose significant threats to the survival and ornamental value of alpine rhododendrons. Although *R. moulmainense* has not yet been extensively reported as suffering from specific diseases or insect infestations, understanding the biotic stress profiles of related alpine species can provide crucial references for early detection, targeted prevention, and ecologically sound control strategies.

Currently, reports have identified the occurrence of diseases on *R. delavayi*, *R. agastum*, *R. irroratum*, *R. decorum*, and *R. parvifolium.* And these diseases mainly occur in rainy and humid weather (Cun et al., 2023; Hu et al., 2022; Pintos et al., 2011; Ren, 2019; Wang et al., 2024; Yang et al., 2019, 2015). The main diseases reported on alpine rhododendrons include stem rot, anthracnose, ulcer disease, leaf blight, gray mold, brown spot disease, black spot disease, root rot disease, wilt disease, and so on. The stem rot is caused by the *Neofsicoccum parvum*, and show the stem turns brown and gradually rots, stem rot (Yang et al., 2015). Anthracnose, caused by *Colletotrichum boninense*, initially manifests as nearly circular brown lesions on the leaf tips or edges of infected rhododendrons. As the infection progresses, these lesions develop black dots, with a grayish-white center and dark brown margins, forming a distinct boundary between diseased and healthy tissue (Yang et al., 2019). The ulcer disease is caused by *Botryosphaeria*, and the symptom include cracking of the epidermis of the branches, decay of the phloem, scattered brown or black spots and tumors on the branches (Ren, 2019).The leaf blight disease is caused by the *Phytophthora*, and shows wilting and discoloration at the base of the stem, yellowing of the leaves of susceptible plants, and the spread of brown lesions from the leaf edges to the base, ultimately leading to complete leaf death and shedding (Žerjav et al., 2004; Rytkönen et al., 2012).

Moreover, a number of pests are known to infest alpine rhododendrons, including aphids, web bugs, red spiders, grubs, shell insects, scarab beetles, and spotted diamond armyworm (Ding, 2022; Xie et al., 2018). Aphid infestations typically occur from March to April and again from November to December, primarily targeting tender leaves and flower buds, resulting in leaf wrinkling and deformation (Ding, 2022; Xie et al., 2018). Web bugs primarily affect leaves, causing rust-yellow discolouration on the back and white spots on the front, and the leaves may turn pale in severe cases (Xie et al., 2018). The red spider occurs from July to September, attacking the undersides of leaves and causing chlorosis and yellowing, which can ultimately lead to plant death (Ding, 2022; Xie et al., 2018). The grubs occur mainly from August to November, and primarily harm the roots, leading to leaf drooping, yellowing, water loss, and in extreme cases, hollowing of the stem pith, which can result in plant mortality (Xie et al., 2018). The scarab beetles occur mainly from May to July, and primarily harm young and tender leaves, causing leaf defects (Xie et al., 2018). Additionally, symptoms of aphid-induced leaf wrinkling have already been observed in natural populations of *R. moulmainense*. Consequently, further identification and comprehensive investigation of diseases and pests is required for establishing effective and environmentally sustainable prevention and control strategies.

These biotic stress factors, although not yet comprehensively documented in *R. moulmainense*, merit focused investigation due to their potential impact on plant health. Despite increasing awareness, molecular mechanisms underlying biotic stress resistance in *R. moulmainense* remain largely unexplored. There is an urgent need to identify resistance-related genes, receptor-ligand interactions, and transcriptional regulators that mediate pathogen recognition and defense activation (Devanna et al., 2021; Jones et al., 2024). *Rhododendron* exemplifies an emerging model for alpine ornamental species capable of ecological plasticity (Liu et al., 2024). Strengthening its disease and pest resistance via molecular breeding or integrated ecological management may further expand its applicability in diversified landscape and restoration contexts.

## 4 The cultivation and reproduction of *Rhododendron moulmainense*

Developing effective propagation and cultivation techniques for *R. moulmainense* is fundamental to its broader utilization in urban landscaping, conservation, and ornamental horticulture. As a representative alpine evergreen rhododendron, *R. moulmainense* exhibits low fruit set under natural conditions and limited autonomous reproductive capability, primarily relying on cross-pollination for generative propagation (Bai, 2017; Ng and Corlett, 2000). Seed propagation is time-intensive, requiring 3-4 years for flowering initiation (Zhang et al., 1994), and germination rates remain variable due to physiological dormancy and environmental constraints.

**Figure 5:**
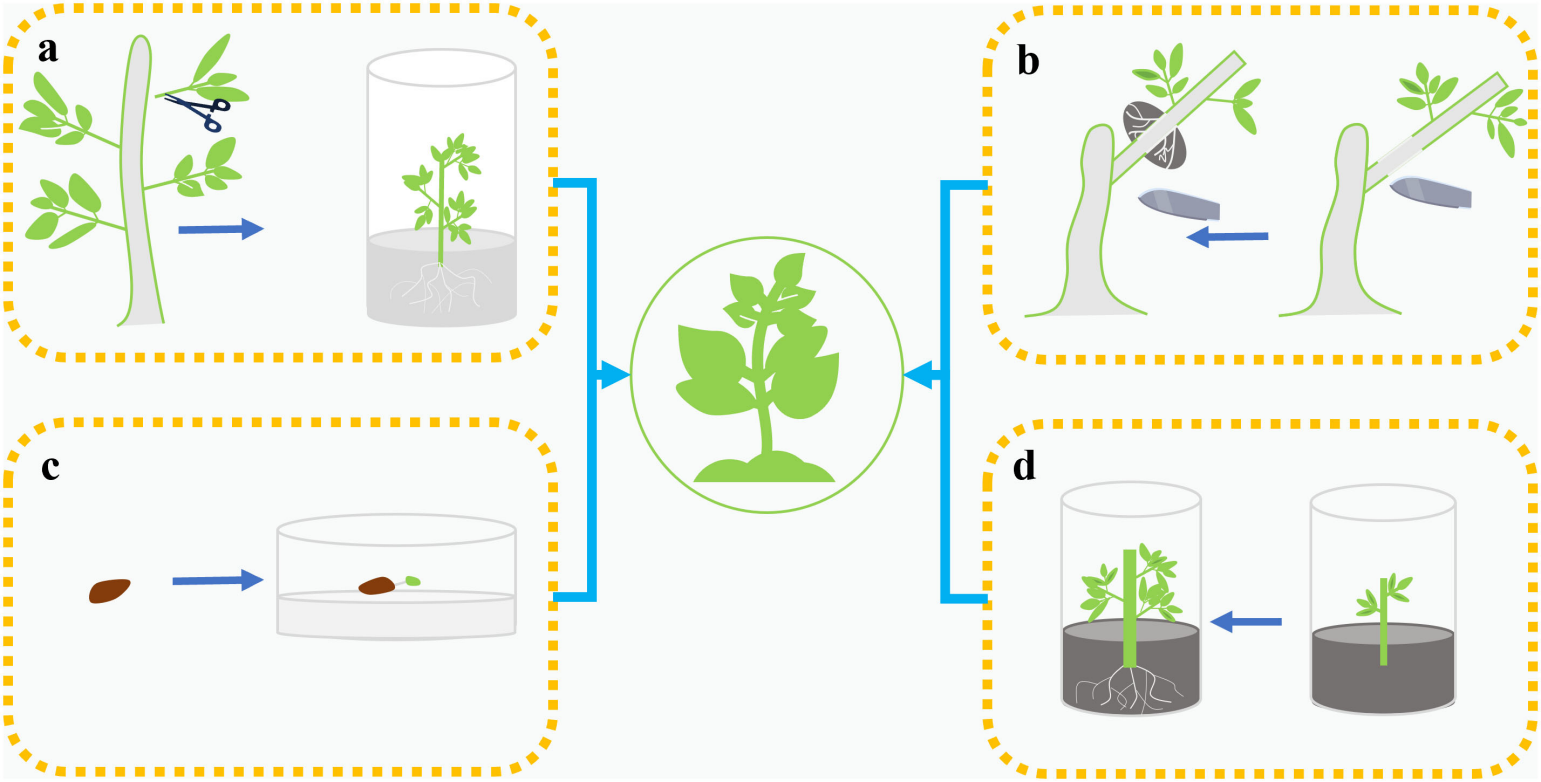
Four propagation methods of *Rhododendron moulmainense*: **(a)** Tender stem tissue is cultured to induce root formation and then nurtured into seedlings. **(b)** For air layering, the sphagnum moss is used to encapsulate the girdled branches, and induce root formation, which is then nurtured into seedlings. **(c)** Seeds are cultivated to grow into seedlings. **(d)** Stem segments are rooted through cuttings and then nurtured into seedlings.

Currently employed propagation strategies include cuttings, juvenile stem culture, air layering, and seed-based systems (**Figure 5**, **Table 3**) (Deng et al., 2022; Ding et al., 2016; Kang et al., 2009; Kochieva et al., 2002; Li and Jia, 2012; Xiong et al., 2011). Experiments using semi-lignified branches of *R. moulmainense* as materials for rooting have shown that a substrate of peat: perlite (1:1) can achieve the best rooting effect (Deng et al., 2022). In addition, a tissue culture system was established using young stems of *R. moulmainense* on basic culture medium (Woody Plant Medium, WPM). Proliferation culture medium (WPM+ZT 2.0 mg·L^-1^+NAA 0.5 mg·L^-1^+Sucrose 30 g·L^-1^, pH 5.0), elongation and robust seedling culture medium (WPM+ZT 0.5 mg·L^-1^+GA_3_ 2.0 mg·L^-1^+Sucrose 30 g·L^-1^, pH 5.0), rooting culture medium (WPM+IAA 0.1 mg·L^-1^+Sucrose 1 g·L^-1^, pH 5.0), and transplanting substrate (perlite: peat soil=1:1) were also used. The cultivation cycle for *R. moulmainense* is 280 days, resulting in a survival rate of over 90% after transplantation (Bai et al., 2020). Zhao et al. developed a seed-based propagation and cultivation system for *R. moulmainense*, using seeds as explants for tissue culture and rapid propagation experiments (Zhao et al., 2017).

**Table 3:** Propagation methodologies for *Rhododendron moulmainense*.

Propagation methods	Primary treatment protocols	Advantages	Limitations	References
Air Layering	Rooting induction via sphagnum moss encapsulation on girdled branches of vigorously growing plants (1.5 cm diameter).	Preservation of maternal genetic traits with induction of precocious flowering.	Labor-intensive operations, low operational efficiency, seasonal constraints, and unsuitability for large-scale multiplication.	(Li and Jia, 2012)
Cutting Propagation	Rooting induction using semi-hardwood cuttings treated with ABT-1 rooting powder via basal immersion, inserted into a peat:perlite (1:1) substrate.	Enhanced lateral root development and higher transplant survival rates, with significantly greater propagation efficiency compared to air layering.	Higher root rot incidence, stringent environmental requirements, and inferior stress tolerance compared to air layering.	(Deng et al., 2022)
Shoot Tip Culture	*In vitro* propagation utilizing young stem explants cultured on WPM basal medium for multiplication, shoot elongation, and root induction.	Season-independent propagation with exceptional clonal fidelity.	Protracted propagation cycles, maternal plant vigor compromised by shoot collection.	(Bai et al., 2020)
Seed Tissue Culture	Seed explants germinated on sucrose-agar medium were subjected to radicle excision upon development of 3-4 true leaves, followed by subculturing on WPM basal medium, multiplication on Read medium, and root induction on 1/2-strength WPM medium.	Seed-derived propagation offers readily accessible explants, promotes well-developed root systems, making it particularly suitable for *ex situ* conservation of critically small populations.	Clonal uniformity is lower and flowering is delayed compared to propagation via shoot tip culture.	(Zhao et al., 2017)
Seedling Propagation via Germination	Seed treatment via GA_3_ immersion significantly enhances germination rates.	Enhanced stress tolerance, well-developed root systems, and lower production costs make this approach particularly suitable for breeding novel cultivars.	Fresh seeds exhibit higher germination rates, whereas stored seeds show significantly reduced germination potential, accompanied by retarded seedling growth, markedly delayed flowering, and failure to retain maternal elite traits.	(Ding et al., 2016)

Seed-based propagation, while still under refinement, offers promising avenues for genotype preservation and the generation of stress-resilient cultivars. Low-temperature storage extends seed viability, whereas exogenous gibberellin application significantly enhances germination rates (Ding et al., 2016). Optimizing substrate ratios, such as peat:sand:perlite at 3:2:1, improves post-transplant survival and morphological uniformity (Song and Zhuang, 2015). Additional environmental studies indicate that *R. moulmainense* seedlings under different shading and soil drainage conditions have demonstrated that the growth is most effective under 50% shading and high ridges (Bai et al., 2017). In addition, studies on planting *R. moulmainense* in different water and light environments on Wutong Mountain in Shenzhen have also shown that *R. moulmainense* thrives in semi-aquatic and semi-light natural environments (Zhang Y., 2022).

Although research on seed propagation-related technologies for *R. moulmainense* is still immature, seed propagation offers many advantages such as well-developed seedlings with strong environmental adaptability, strong stress resistance. Nonetheless, several challenges remain. Seed propagation techniques are still at the exploratory stage, and the species’ long developmental cycle necessitates further optimization of storage parameters, germination enhancers, and substrate formulations (Bai et al., 2020; Xiong et al., 2011). On the molecular front, research should explore genetic markers associated with rooting efficiency, stress tolerance, and flowering onset to inform precision breeding and propagation (Li and Jia, 2012; Xiong et al., 2011). In particular, integrating transcriptomic and metabolomic data into cultivation protocols could help identify physiological bottlenecks and accelerate phenotypic screening for adaptable varieties.

## 5 Conclusion and future perspectives

**Figure 6:**
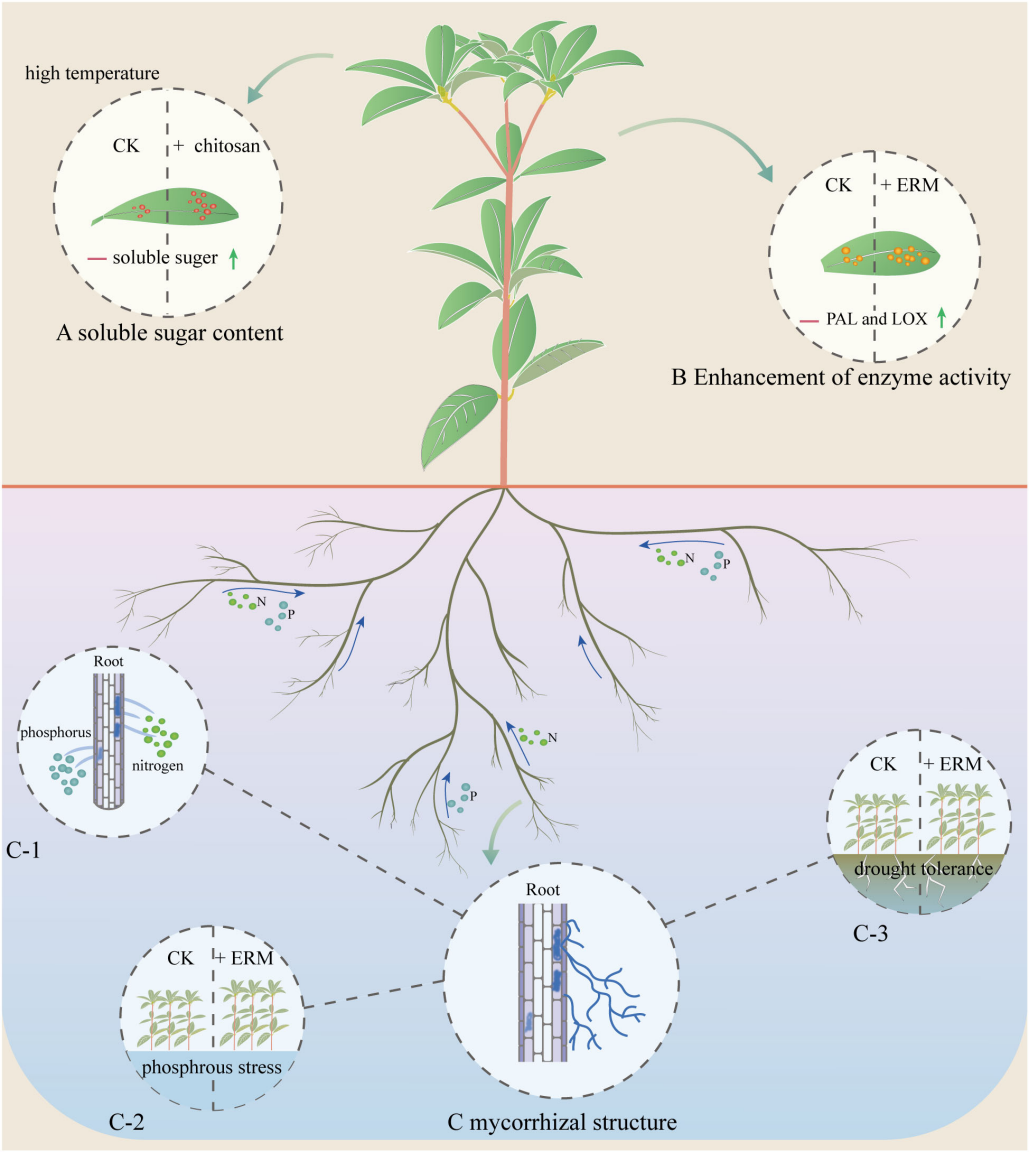
Role of mycorrhizal fungi and chitosan on nutrient uptake, growth, and response to adversity stress in the root system of *Rhododendron moulmainense*. **(A)** Increase in leaf soluble sugars by exogenous substance chitosan application to *R. moulmainense* leaves under high temperature conditions, **(B)** Fungal inoculation of *R. moulmainense* roots increases leaf active enzymes PAL and LOX content, **(C)** mycorrhizal structure, C-1. the root system is inoculated with fungi such as *Phialocephala fortinii*, *Aspergillus sydowii*, *Bionectria ochroleuca* and *Paecilomyces javanicus*. This enhances the adaptability of *R. moulmainense* to adverse environmental conditions and promotes the absorption of elements, thereby facilitating the growth of *R. moulmainense*, C-2. Fungal inoculation of *R. moulmainense* improves phosphorus acclimatisation for growth under phosphorus stress, C-3. Fungal inoculation of *R. moulmainense* to improve drought adaptation and growth under drought tolerance.

Research on *R. moulmainense* has progressively expanded across multiple domains, including phylogenetic analysis, breeding and propagation technologies, photosynthetic physiology, stress resistance, and mycorrhizal symbiosis (**Figure 6**). These foundational studies not only deepen our understanding of this alpine evergreen species but also provide essential references for its conservation, domestication, and ecological utilization in lower-altitude urban contexts.

Despite these advances, considerable challenges remain in bridging fundamental research with practical application. Propagation techniques, especially seed-based and air layering, remain largely experimental and have not been widely adopted for scalable cultivation. Seed germination is impeded by physiological dormancy, long developmental cycles, and low survival rates post-transplantation (Bai et al., 2020; Xiong et al., 2011). While tissue culture and cutting propagation have achieved primary success (Li and Jia, 2012; Xiong et al., 2011), further refinement is required to improve efficiency and reduce cost. Stress resistance studies have thus far focused predominantly on high-temperature responses, including antioxidant regulation and molecular thermotolerance mechanisms (Liu et al., 2024; Wang et al., 2021). However, future climate variability may expose *R. moulmainense* to a broader range of abiotic and biotic pressures, including drought, fluctuating humidity, pest infestation, and urban pollutants. Moreover, light not only drives photosynthesis but also modulates temperature perception and bud differentiation (Xie et al., 2010; Zhang et al., 2023), indicating a need to consider interactive environmental effects. The role of ericoid mycorrhizal fungi is particularly critical for *R. moulmainense*, given its alpine origin and reduced nutrient availability in urban soils. Although research has identified diverse fungal communities associated with its root system (Gong et al., 2021; Peng et al., 2022), the functional mechanisms underlying microbial-mediated growth enhancement remain poorly understood (Liu et al., 2015a; Song et al., 2015; Zhou et al., 2017a, 2017b). Questions persist regarding how fungal symbionts influence soil structure, nutrient bioavailability, and stress mitigation, as well as the biochemical feedback between plant metabolites and rhizosphere composition. Targeted isolation and cultivation of beneficial fungi, potentially as organic bio-stimulants, may help overcome acclimatization challenges following introduction to lowland regions. Flowering physiology is another promising yet underexplored avenue. *R. moulmainense*’s vibrant floral displays enrich urban landscapes. Current studies have focused on bud differentiation and hormonal cues (Xie et al., 2010; Zhang et al., 2023), but further exploration into flowering duration, season extension, and metabolite utilization could unlock new ornamental and commercial applications. Breeding programs aimed at prolonging bloom periods or enhancing floral stability under stress would be particularly impactful.

In summary, *R. moulmainense* provides a valuable model for developing stress-resilient ornamental plants suited to eco-sensitive urban and restoration landscapes. As climate pressures intensify and public demand for sustainable greenery increases, advancing research in propagation, stress physiology, microbial interactions, and floral development will be essential for realizing its full ecological and aesthetic potential.
